# Which Safe Zone Is Safe in Total Hip Arthroplasty? The Effect of Bony Impingement

**DOI:** 10.3390/jpm12050812

**Published:** 2022-05-18

**Authors:** Markus Weber, Frederik von Kunow, Moritz Innmann, Matthias Meyer, Max Thieme, Seth Jerabek, Tobias Renkawitz

**Affiliations:** 1 Faculty of Medicine, University of Regensburg, 93053 Regensburg, Germany; 2 Department of Orthopedic and Trauma Surgery, Barmherzige Brueder Regensburg Medical Center, 93049 Regensburg, Germany; frederik.vonkunow@barmherzige-regensburg.de; 3 Department of Orthopaedics, Heidelberg University Hospital, 69120 Heidelberg, Germany; moritzinnmann@gmail.com (M.I.); tobias.renkawitz@med.uni-heidelberg.de (T.R.); 4 Department of Orthopedic Surgery, University of Regensburg, 93077 Bad Abbach, Germany; matthias.meyer@klinik.uni-regensburg.de (M.M.); maxthieme1@gmail.com (M.T.); 5 Orthopaedic Surgery, Adult Reconstruction and Joint Replacement, Hospital for Special Surgery, New York, NY 10021, USA; jerabeks@hss.edu

**Keywords:** total hip arthroplasty, bony impingement, combined anteversion, safe zone

## Abstract

“Safe zones” for cup position are currently being investigated in total hip arthroplasty (THA). This study aimed to evaluate the impact of bony impingement on the safe zone and provide recommendations for cup position in THA. CT scans were performed on 123 patients who underwent a cementless THA. Using the implant data and bone morphology from the CT scans, an impingement detection algorithm simulating range of motion (ROM) determined the presence of prosthetic and/or bony impingement. An impingement-free zone of motion was determined for each patient. These zones were then compared across all patients to establish an optimized impingement-free “safe zone”. Bony impingement reduced the impingement-free zone of motion in 49.6% (61/123) of patients. A mean reduction of 23.4% in safe zone size was observed in relation to periprosthetic impingement. The superposition of the safe zones showed the highest probability of impingement-free ROM with cup position angles within 40–50° of inclination and 20–30° of anteversion in relation to the applied cup and stem design of this study. Virtual ROM simulations identified bony impingement at the anterosuperior acetabular rim for internal rotation at 90° of flexion and at the posteroinferior rim for adduction as the main reasons for bony impingement.

## 1. Introduction

Although total hip arthroplasty (THA) is one of most successful procedures in orthopaedic surgery, with satisfaction rates over 90% [1], surgeons continue to be challenged by impingement and stability. In addition to restoring the biomechanics of the hip through hip length and offset, accounting for patient-specific factors such as bony morphology and the 3D position of the components may improve the range of motion (ROM) prior to impingement and provide better stability and function. The fact that more than half of the retrieved acetabular components show signs of impingement [2] emphasizes the importance of this issue. In addition, instability and dislocation are some of the most common causes of revision total hip arthroplasty, accounting for 22.5% of all revisions [3]. However, the optimal cup position angles remain unclear. The traditional thinking of a universal safe zone is too simplistic. It is well established that cup positioning within the Lewinnek safe zone does not prevent dislocation [4,5]. The concept of combined anteversion has highlighted the importance of the relationship between the cup and stem version and range of motion [6]. In the literature, a variety of different recommendations for combined anteversion have been described. However, none of the current available tools are fully able to avoid bony impingement [7]. In the current study, we intended to establish an optimized cup position zone that minimizes implant and bony impingement.

Therefore, we questioned whether bony impingement is relevant for free ROM, as well as which combinations of cup anteversion and inclination enable free ROM without impingement using 3D-CT ROM simulations in 123 patients after THA. Second, we analyzed the causes of failure and detected relevant impingement areas focusing on the interaction of acetabulum/cup and femur/stem, and not accounting for soft tissue impingement.

## 2. Patients and Methods

During a prospective controlled trial (DRKS00000739, German Clinical Trials Register), three-dimensional computed tomography scans (3D-CT) were performed after cementless THA. The main outcome of the study compared the accuracy of conventional and navigation-guided THA [8]. The current study is an independent secondary outcome analysis. A sovereign power calculation was performed for the primary endpoint “reduction of safe zone size due to bony impingement” in this analysis on a two-sided test with a 5% significance level. Based on previous calculations [7], the effect size was conservatively estimated to be 0.4 for two dependent variables. Using these considerations, a total sample size of 68 achieved a power of 90% using two-sample t-tests for dependent parameters (G*Power 3.1, Düsseldorf, Germany). The investigation was approved by the medical ethics committee of the University of Regensburg (no. 10-121-0263, date of approval: 19.04.2011).

**Figure 1:**
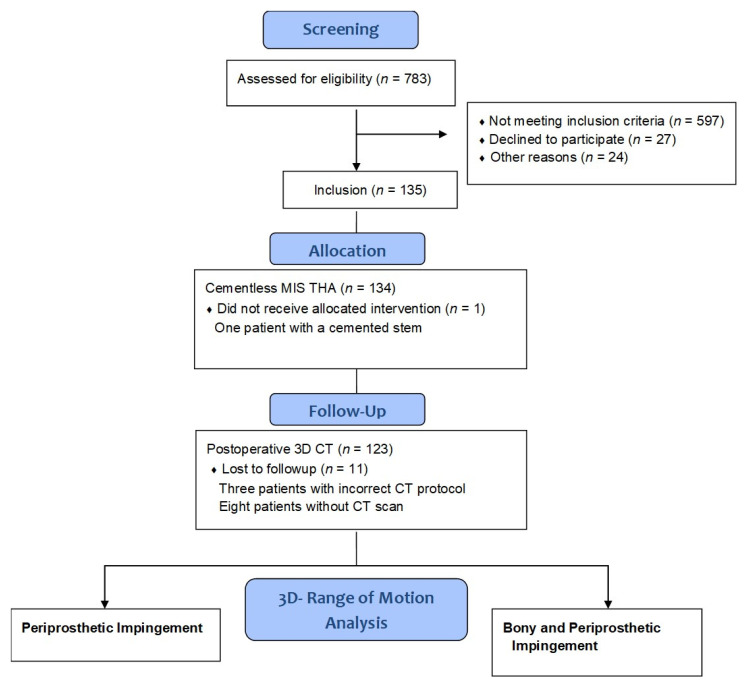
Flow diagram of the participants in this study. MIS THA, minimally-invasive total hip arthroplasty; 3D CT, three-dimensional computed tomography.

According to the study protocol, eligible participants were patients between the ages of 50 and 75 with an American Society of Anaesthesiologists (ASA) score ≤3 who were admitted for primary cementless unilateral THA due to primary or secondary osteoarthritis at our institution. No patients had significant disease in the contralateral hip. Exclusion criteria were age <50 (to avoid radiation damage as a post-operative CT scan was required) and >75 years (to ensure post-operative long-term follow up was achieved), ASA score >3, arthritis of the secondary to hip dysplasia, post-traumatic hip deformities, and previous hip surgery. Patients were recruited and informed consent was obtained by one of the clinical investigators. Figure 1 summarizes the data on the participants in the study. THA in all patients was performed in the lateral decubitus position using a minimally-invasive single-incision anterolateral approach [9]. Press-fit acetabular components with neutral polyethylene liners and cement-free hydroxyapatite-coated stems (Pinnacle cup, Corail stem, DePuy, Warsaw, IN, USA) with metal heads of 32 mm were used, except for one case with severe osteoporosis where the stem was cemented. Standard or high offset geometry of the stem was chosen according to the patient’s native femoral offset. Because of the elliptic neck design of the stem, the head neck ratio was 3.50 for extension/flexion (anteroposterior direction) and 2.66 for abduction/adduction (mediolateral direction). Six weeks postoperatively, a pelvic/femoral 3D-CT was performed including imaging through the knee to determine femoral version (Somatom Sensation 16; Siemens, Erlangen, Germany).

In total, 123 data sets were included for analysis. The anthropometric characteristics of the study group are shown in Table 1. Manual CT segmentation was performed on the pelvic bone and on the metal acetabular and femoral components by an independent external institute (Fraunhofer MEVIS, Bremen, Germany), blinded to individual patient data. Cup inclination, anteversion, and stem antetorsion were evaluated by the independent external institute on the manually segmented reconstruction of the pelvis and femur using image-processing software (based on MeVisLab, MeVis, Bremen, Germany), as previously described [10,11,12,13,14]. Based on the manually segmented bone models, the postoperative ROM was calculated using a previously evaluated algorithm that automatically determines single prosthetic or combined bony and prosthetic impingement by virtually moving the leg until a collision between the 3D objects occurs [13,15]. Neutral orientation for ROM calculations was defined according to the anterior pelvic plane (APP) with the femur mechanical axis parallel to the APP and sagittal plane and foot directed straight forward without rotation. The high accuracy of this collision detection algorithm was demonstrated in a previous study [7]. This model with the given antetorsion of the implanted stem was run multiple times for each patient virtually by the varying cup angles, but not the center of rotation, ranging from 10° to 60° for inclination and 0° to 50° for anteversion referring to the radiographic definition and APP, respectively. For all cup position angles, the ROM analysis was performed twice; the first time without the osseous information only assessing periprosthetic impingement and the second time including both prosthetic and bony information. We then assessed the number of cup positions reaching the modified hip joint ROM configurations without impingement for activities of daily living (ADL) given the recommendations by Davis et al., Miki et al., and Turley et al. with at least 100 degrees of flexion, 20 degrees of extension, 40 degrees of abduction, 20 degrees of adduction, 30 degrees of external rotation during extension, and 20 degrees of internal rotation during 90 degrees of hip flexion, respectively [16,17,18]. These results were used to generate a safe zone for cup positions achieving all ROM criteria as originally described by Widmer et al. [6]. We then assessed the position and size of the safe zones, differentiating between exclusive prosthetic and combined osseous and prosthetic impingement. The corresponding safe zone size was computed as the surface of a sphere using a radius of one. The sphere represents all possible cup position angles. Furthermore, the center of the safe zones was calculated and described with the corresponding inclination and anteversion angle. Based on the information, a general model was developed to provide recommendations for general best possible cup position angles in THA. In addition, further impingement-related confounders such as femoral offset and neck length restoration were computed in comparison with the unaffected contralateral side [19].

For statistical analysis, normally and nonnormally distributed continuous data are presented as mean (standard deviation) or median (range), respectively. Accordingly, group comparisons were performed using two-sided t-tests or Mann−Whitney U-tests with a 5% significance level. Absolute and relative frequencies were given for categorical data and compared between groups using Chi-square tests with a 5% significance level. IBM^®^ SPSS^®^ Statistics 25 (IBM, Armonk, NY, USA) was used for analysis.

## 3. Results

**Figure 2:**
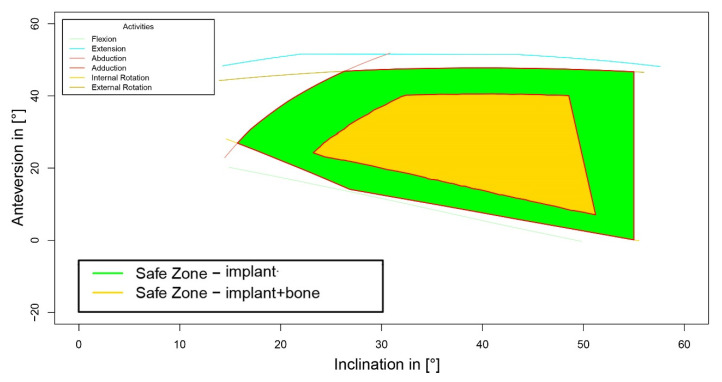
Reduction in safe zone due to bony impingement.

After analyzing the impact of bony impingement on the size of a cup safe zone, the additional information of bony structures showed a reduction in the calculated safe zones in 49.6% (61/123) of patients. Quantifying the size of reduction of possible cup position angles for free ROM using a sphere model (as described above), the safe zone size decreased from 1.4 ± 0.1 for periprosthetic impingement to 1.1 ± 0.6 (*p* < 0.001, Figure 2). This resulted in a ratio of combined bony/periprosthetic to single periprosthetic safe zone size of 76.6 ± 40.5%. In contrast, the center of the safe zones did not change, and was clinically irrelevant with −0.1° ± 0.4° (*p* < 0.001) for cup inclination and 0.2° ± 0.7° (*p* < 0.001) for cup anteversion, respectively.

**Figure 3:**
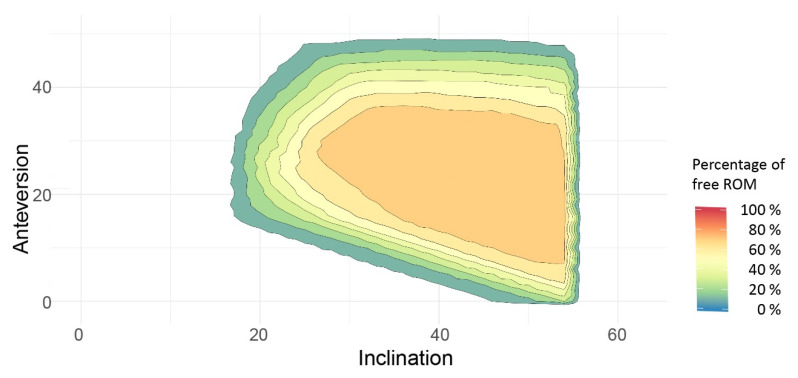
Superposed combined safe zones of the study cohort. ROM, range of motion.

Based on all of the available information, the safe zones for combined bony and/or prosthetic impingement were superposed to establish the best possible cup position angles enabling free ROM, referring to the ROM values mentioned in the Patients and Methods section in the majority of patients. The data showed the highest probability of free ROM if the cup was placed within 40–50° of inclination and 20–30° of anteversion (Figure 3).

**Figure 4:**
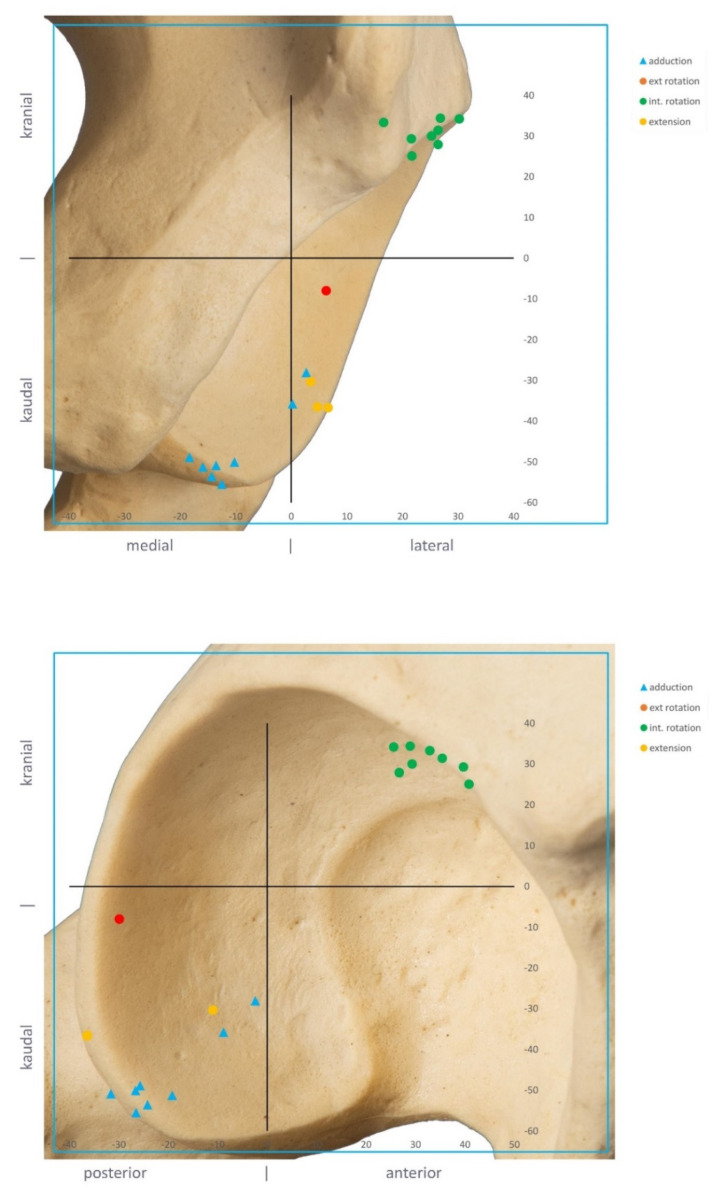
Bony impingement areas at the acetabulum as the cause of failure.

We investigated the bony impingement areas in cases with a severe reduction in safe zone size. Using the segmented 3D models, the anatomical correlate could be detected and visualized. The ROM simulations identified bony impingement at the anterosuperior acetabular rim for internal rotation at 90° flexion and at the posteroinferior rim for adduction as the reason for bone-to-bone or bone-to-implant impingement in these cases (Figure 4).

During clinical follow-up, we experienced one dislocation after surgery. Although the cup position was within the Lewinnek safe zone, the 3D-CT analysis revealed a bony impingement at the anterosuperior rim.

## 4. Discussion

Despite the high success rate of THA, impingement and instability remain a frequent cause of failure [3]. As historical targets for optimal cup position, such as the Lewinnek safe zone, are not clinically predictive of stability [4,5], future studies have to account for more variables that may be more predictive. This work evaluates the role of combined anteversion and bony morphology on bony impingement after THA. We aimed to analyze the effect of bone on combined anteversion safe zones and to evaluate the causes of failure. Because of bony impingement, cup safe zones were reduced in 50% of patients in our cohort. Impingement occurred for flexion at 90° flexion at the anterosuperior acetabular rim and for adduction at the posteroinferior rim. Cup position angles within 40–50° of inclination and 20–30° of anteversion showed the highest probability of free ROM.

There are several limitations of this study. First, the 3D-CT impingement detection algorithm assessed osseous and prosthetic impingement. However, we were not able to look for soft-tissue-related impingement. In obese patients, soft tissue may limit ROM before hardware impingement occurs. Additionally, iliopsoas impingement is a further parameter influencing ROM [20]. Second, we performed only cementless THA through a minimally invasive anterolateral approach using non-modular components of one manufacturer (femoral neck shaft angle 135°, cone 12/14, head diameter 32 mm, head/neck ratio 3.50 for extension/flexion and 2.66 for abduction/adduction). Therefore, the results might differ for other prosthetic designs. Third, our 3D-CT-based assessment of the cup inclination and version was performed referencing the APP without adjusting for functional pelvic positions. Pelvic tilt can change during gait and provocative positions such as sitting or squatting [21]; thus, it is a dynamic process that may affect impingement and stability. However, it is the authors’ opinion that the topic of pelvic tilt and functional THA positioning has not been fully evaluated or sufficiently studied to determine its contribution at this time. Fourth, CT scans were performed after surgery. The vast majority of osteophytes was removed during THA. However, it cannot be ruled out that additional bone had remained. Therefore, it was challenging to differentiate between osteophytes and original acetabular rim.

A strength of the study is the use of actually implanted cases and not just simulation through virtual 3D planning. Therefore, the results reflect the clinical practice and real position of the implants in the body. In addition, possible confounders limiting ROM such as leg length, offset, and center of rotation were controlled [19].

In response to the first question of the study, we were able to demonstrate the importance of bony information for ROM. Bony impingement led to a reduction in the safe zone size regarding possible cup positions in half of the patients, thus narrowing the intraoperatively desired target area for cup position angles. Quantifying the size of reduction in safe zone size indicated that it was decreased by approximately 25% as a result of bony impingement. However, this change was highly variable, as shown by the high standard deviation. Interestingly, the safe zone decreased symmetrically, leaving the center of the safe zone mostly untouched, with mean differences below one degree for both cup inclination and anteversion, which can be regarded as clinically irrelevant. In this context, current available definitions of combined anteversion rules should be used with caution as they focus on periprosthetic impingement and do not account for bony impingement [6,22].

Regarding recommendations for cup position angles, the Lewinnek safe zone is still widely applied among orthopaedic surgeons. However, the literature shows that 58% of dislocated cups are within the Lewinnek safe zone, challenging the safety of this so-called safe zone [4]. On the other hand, modern technologies such as navigation and robotics harbor the possibility for intraoperative ROM analysis using impingement detection algorithms, but are not always available and involve some expense [13]. Therefore, feasible and valid recommendations for cup positioning angles are required for routine use. To further analyze this challenging issue, we superposed the safe zones in our cohort and found the highest probability for high ROM if the cup was placed within 40–50° of inclination and 20–30° of anteversion. In contrast with the Lewinnek propagated safe zone, the required ROM of 15° of cup anteversion is only achievable if the cup inclination is at least 40° or more. In relation to Lewinnek, we advise a higher cup anteversion [23]. Remarkably, the propagated novel safe zone was not able to prevent bony impingement in all cases either.

When researching the causes of impingement, we identified bony impingement areas at the anterosuperior rim for internal rotation at 90° of flexion and at the posteroinferior rim for adduction as the main reasons for limited ROM. As a consequence, surgeons are encouraged to thoroughly remove osteophytes and ossifications at these areas after implantation of the final cup in order to prevent impingement. Although a combined anteversion of the cup and stem [6] or even patient-specific target zones [24] might still be preferable, this current recommendation represents a feasible orientation for cup positioning angles, despite the high range of stem antetorsion in cementless stem designs.

In conclusion, because of bony impingement, safe zones for cup positions are smaller than initially calculated in the literature. As a general recommendation, cup inclination between 40 and 50° and cup anteversion between 20 and 30° seem to show the highest safety towards impingement regarding the implants used in this study.

## Figures and Tables

**Table 1:** Anthropometric and operative characteristics of the study group.

Sex (female)	53.7% (66/123)
Age (years)	62.6 ± 7.6
BMI (kg/m^2^)	27.1 ± 4.2
Treatment side (right)	54.5% (67/123)
ASA 1	20.3% (25/123)
ASA 2	51.2% (63/123)
ASA 3	28.5% (35/123)
Kellgren-Lawrence-Score	8 (5–10)
Cup size	54 (48–62)
Femoral component size	12 (9–16)
Cup inclination (°)	42.4 ± 5.8
Cup anteversion (°)	17.9 ± 8.0
Stem antetorsion (°)	8.0 ± 9.5
Femoral Offset (mm)	47.0 ± 4.9
Neck length (mm)	44.5 ± 4.2
Operation time (minutes)	67.5 ± 13.8

BMI = body mass index; ASA = American Society of Anaesthesiology Score. For categorical data, values are given as relative and absolute frequencies; for quantitative data, values are given as mean ± standard deviation or median (range).

## Data Availability

The data presented in this study are available on request from the corresponding author. The data are not publicly available due to medical reasons.
